# Otologische Prävention – Schlüssel zu lebenslanger Hörgesundheit

**DOI:** 10.1007/s00106-026-01741-5

**Published:** 2026-02-17

**Authors:** Paul Emmerich Krumpoeck, Lukas David Landegger

**Affiliations:** 1 Christian-Doppler-Labor für Innenohrforschung, Universitätsklinik für Hals-, Nasen- und Ohrenheilkunde, Kopf- und Halschirurgie, Medizinische Universität Wien, Währinger Gürtel 18–20, 1090 Wien, Österreich; 2 Allgemeines Krankenhaus Wien, Universitätsklinik für Hals-, Nasen- und Ohrenheilkunde, Kopf- und Halschirurgie, Medizinische Universität Wien, Wien, Österreich; 3 Department of Otolaryngology—Head and Neck Surgery, Stanford University School of Medicine, Stanford, CA USA

**Keywords:** Hörverlust, Präventivmedizin, Diagnostische Screeningprogramme, Impfstoffe, Korrektur von Hörbeeinträchtigungen, Hearing loss, Preventive medicine, Diagnostic screening programs, Vaccines, Auditory rehabilitation

## Abstract

**Hintergrund:**

Hörverlust ist ein weltweites Gesundheitsproblem mit potenziell schwerwiegenden Folgen für Sprachentwicklung, soziale Integration und kognitive Fähigkeiten. Ein erheblicher Teil dieser Belastung lässt sich durch gezielte Strategien verhindern.

**Ziel der Arbeit:**

Die vorliegende Literaturübersicht fasst wichtige evidenzbasierte Präventionsmaßnahmen in der Otologie zusammen und gibt Empfehlungen für die Praxis.

**Material und Methoden:**

Es erfolgte eine kritische Bewertung und Zusammenfassung aktueller nationaler klinischer Leitlinien, systematischer Übersichtsarbeiten sowie relevanter epidemiologischer und klinischer Studien.

**Ergebnisse:**

Wichtige Präventionsmaßnahmen beginnen bereits vor der Geburt mit Impfungen und Hygieneberatung für Schwangere sowie Vorsorgeuntersuchungen wie Screening auf Syndrome oder kongenitale Zytomegalievirus(CMV)-Infektion. Das universelle Neugeborenen-Hörscreening ist ein Eckpfeiler für frühzeitige Diagnose und Intervention und ermöglicht optimierte Ergebnisse mit Cochleaimplantaten oder neuartigen Gentherapien. Impfungen für Kinder, Lärmschutz, limitierter Einsatz ototoxischer Medikamente und Optimierung lebensstilbezogener Faktoren sind wirksame Strategien zur Prävention von erworbenem Hörverlust. Darüber hinaus ist die auditive Rehabilitation mit Hörgeräten oder Implantaten entscheidend für die Tertiärprävention, um Folgen wie soziale Isolation und kognitiven Verfall zu mildern.

**Schlussfolgerung:**

Von der Beratung werdender Eltern bis hin zur Sicherstellung einer rechtzeitigen Rehabilitation bei älteren Erwachsenen spielen HNO-Ärzte eine zentrale Rolle bei der Umsetzung vielschichtiger, proaktiver Präventionsstrategien zur Förderung der Hörgesundheit und zur Verminderung der Belastung durch Schwerhörigkeit.

## Abstract (en)

**Background:**

Hearing loss is a major global health issue with potentially severe consequences for speech development, social integration, and cognitive health. A significant proportion of this burden is preventable through targeted strategies applied across the human lifespan.

**Objective:**

This narrative review synthesizes key evidence-based preventive measures in otology and provides practical recommendations for clinicians.

**Materials and methods:**

A selective review of the current literature was conducted, including national clinical guidelines, systematic reviews, epidemiological studies, and pivotal clinical trials.

**Results:**

Key preventive measures begin before birth with maternal vaccinations and hygiene counseling as well as screening for syndromes or congenital cytomegalovirus infection. Universal newborn hearing screening is a cornerstone of early diagnosis and intervention, enabling superior outcomes with cochlear implantation or emerging gene therapies. Recommended childhood immunizations, noise protection, cautious use of ototoxic medications, and managing lifestyle-related risk factors are effective strategies for preventing acquired hearing loss. Furthermore, auditory rehabilitation with hearing aids or implants is crucial for tertiary prevention, mitigating secondary consequences such as social isolation and cognitive decline.

**Conclusion:**

A multifaceted, proactive, life-course approach to hearing health is essential to reduce the burden of hearing loss. Otolaryngologists play a central role in implementing these preventive strategies, from counseling expectant parents to ensuring timely rehabilitation in older adults.

Hörverlust ist eine der häufigsten Sinnesbeeinträchtigungen und betrifft weltweit über 1,5 Mrd. Menschen. Die Folgen übersteigen die Hörminderung per se und beeinflussen kognitive Gesundheit, soziale Integration und kindliche Entwicklung. Obwohl Hörverlust ein Teil des normalen Alterungsprozesses sein kann, ist ein erheblicher Teil vermeidbar. Diese Arbeit beleuchtet essenzielle Strategien in der Otologie, um das Gehör zu erhalten, von der Schwangerschaftsvorsorge bis hin zu geriatrischen Patienten.

## Inzidenz von Hörverlust

Hörverlust ist weltweit eine der häufigsten Sinnesbeeinträchtigungen. Schätzungsweise 1,57 Mrd. Menschen sind davon betroffen, wobei etwa 430 Mio. einen Hörverlust moderater oder schwerer Ausprägung aufweisen [28]. Die Auswirkungen reichen weit über die auditive Wahrnehmung hinaus und beeinträchtigen Sprachentwicklung, soziale Integration und kognitive Gesundheit [15, 23].

Ein erheblicher Teil der globalen hörverlustassoziierten Gesundheitsbelastung lässt sich verhindern

Jedoch lässt sich ein erheblicher Teil der globalen mit Hörverlust assoziierten Gesundheitsbelastung durch Strategien verhindern, welche sich klassischerweise in 3 verschiedene Ebenen einordnen lassen: Primärprävention, die anstrebt, das Auftreten von Hörverlust gänzlich zu verhindern; Sekundärprävention, die sich auf Früherkennung und Intervention konzentriert, um das Fortschreiten zu stoppen oder zu verlangsamen; und Tertiärprävention, die darauf abzielt, zukünftige Auswirkungen von Hörverlust abzuschwächen. Die folgenden Kapitel halten sich an diese Einteilung und sind chronologisch angeordnet, indem sie evidenzbasierte Maßnahmen von der pränatalen Phase bis hin zum höheren Alter skizzieren. Die wichtigsten präventiven Maßnahmen, die in den einzelnen Lebensphasen üblicherweise eingesetzt werden, sind in Tab. 1 aufgeführt.

**Tab. 1:** Wichtige Präventionsstrategien in der Otologie über die gesamte Lebensspanne hinweg.

Lebensphase	Art der Prävention	Wichtige Maßnahmen
Prä‑/Perinatal	Primär	Maternale MMR-Impfung; CMV-Hygieneberatung
Säuglingsalter	Sekundär	UNHS; gezieltes oder universelles CMV-Screening
Kindheit	Primär	Routineimpfungen (MMR, HiB, Pneumokokken)
	Sekundär/Tertiär	Frühintervention (z. B. CI) bei diagnostiziertem Hörverlust
Erwachsenenalter	Primär	Gehörschutz (Beruf/Freizeit); Ototoxizitätsmonitoring; gesunde Lebensweise (Ernährung, Raucherentwöhnung/Alkoholvermeidung)
	Sekundär	Opportunistische oder systematische Hörscreenings
Höheres Alter	Tertiär	Hörrehabilitation (Hörgeräte/CI)

*CI *Cochleaimplantat,* CMV *Zytomegalievirus,* HiB Haemophilus influenzae *Typ B, *MMR *Masern/Mumps/Röteln, *UNHS* universelles Neugeborenen-Hörscreening

## Prä- und perinatale Prävention

Die Grundlage für ein gesundes Gehör wird bereits vor der Geburt gelegt. Die prä- und perinatale Prävention konzentriert sich auf die Risikominderung angeborener Infektionen, einer der wichtigsten vermeidbaren Ursachen für sensorineuralen Hörverlust (SNHV). Die bekannteste Erfolgsgeschichte ist hier die Prävention des angeborenen Rötelnsyndroms. Eine Infektion der Mutter, insbesondere im ersten Trimester, kann zu einer Trias von Katarakten, Herzfehlbildungen und schwerem beidseitigem SNHV führen [14]. Durch die flächendeckende Einführung der Masern-Mumps-Röteln(MMR)-Impfung ist diese Erkrankung in Ländern mit hoher Impfquote äußerst selten geworden. Die meisten nationalen Leitlinien empfehlen, dass alle Personen, insbesondere Frauen im gebärfähigen Alter, über eine durch 2 Impfdosen dokumentierte Immunität verfügen sollten. Da der MMR-Impfstoff ein abgeschwächtes Lebendvirus enthält, ist er während der Schwangerschaft kontraindiziert, weshalb es entscheidend ist, den Impfstatus bereits bei Kinderwunsch zu überprüfen.

Die kongenitale Infektion mit dem Zytomegalievirus (CMV) ist in Industrieländern die häufigste angeborene Infektion und die führende nichtgenetische Ursache für SNHV bei Kindern mit einer geschätzten Inzidenz von 0,2–0,6 % aller Lebendgeburten in Deutschland. Etwa 10–15 % der infizierten Neugeborenen zeigen bei der Geburt Symptome wie Mikrozephalie oder Petechien, wobei ein hoher Anteil dieser Säuglinge SNHV entwickelt. Die Mehrheit der Neugeborenen mit konnatalem CMV ist asymptomatisch, obwohl 10–15 % dennoch einen spät auftretenden, oft progredienten SNHV entwickeln [9]. Da zzt. kein Impfstoff verfügbar ist, beruht die Primärprävention auf Verhaltensmaßnahmen. Der Hauptinfektionsweg ist die vertikale Übertragung einer Primärinfektion (jedoch nicht einer Reaktivierung) bei seronegativen Schwangeren, häufig durch Kontakt mit Speichel oder Urin von Kleinkindern [14]. Einfache Hygienehinweise, wie z. B. häufiges Händewaschen und das Vermeiden des Teilens von Speisen, Getränken und Besteck mit Kleinkindern, sind eine wirksame Strategie zur Verringerung des mütterlichen Infektionsrisikos [7].

Für die Sekundär- und Tertiärprävention ist die Früherkennung von größter Bedeutung. Das diagnostische Zeitfenster ist eng, da eine angeborene CMV-Infektion durch eine Polymerasekettenreaktion (PCR) von Blut‑, Speichel- oder Urinproben innerhalb der ersten 21 Lebenstage bestätigt werden muss, um eine postnatale Infektion auszuschließen [7]. Obwohl zunehmend befürwortet, ist das universelle Neugeborenen-Hörscreening (UNHS) auf CMV in den meisten Ländern noch nicht Standard. Stattdessen empfehlen die deutschen Leitlinien, Neugeborene nur dann auf CMV zu testen, wenn sie das UNHS nicht bestehen [9, 20].

Eine angeborene CMV-Infektion muss innerhalb der ersten 21 Lebenstage bestätigt werden

Frühzeitige Erkennung ermöglicht eine langfristige audiologische Nachsorge und rechtzeitige Intervention. Bei Neugeborenen mit symptomatischer angeborener CMV-Infektion hat sich gezeigt, dass eine antivirale Behandlung mit Valganciclovir über 6 Monate die langfristigen audiologischen und neurologischen Entwicklungsergebnisse verbessert und das Fortschreiten des Hörverlusts verhindert [7, 13]. Während die Evidenz für die Behandlung asymptomatischer Säuglinge noch vage ist, bleibt die Fähigkeit, eine angeborene CMV-Infektion innerhalb des kritischen neonatalen Zeitfensters zu diagnostizieren, die Voraussetzung für jede therapeutische Entscheidung.

## Früherkennung und Prävention im Kindesalter

Die ersten Lebensjahre sind eine entscheidende Phase für die Entwicklung des Gehörs und des Nervensystems. Daher sind frühzeitige Diagnose und Intervention von größter Bedeutung, um schwerwiegende Folgen einer Hörbeeinträchtigung – insbesondere Defizite in der Sprach‑, Sprech- und kognitiven Entwicklung – zu vermeiden. UNHS-Programme sind ein Eckpfeiler der pädiatrischen Audiologie und gelten in vielen Ländern als Standardverfahren, darunter seit 2009 in Deutschland und seit 2003 in Österreich [20]. Die Programme folgen dem internationalen „1-3-6-Schema“: Screening im Alter von 1 Monat, Diagnose im Alter von 3 Monaten und Intervention im Alter von 6 Monaten [12]. Die angewandten Methoden sind sehr sensitiv (fast 100 %), objektiv und nichtinvasiv, wodurch das mediane Diagnosealter erfolgreich gesenkt werden konnte. Die meisten Säuglinge werden zunächst mithilfe von transitorisch evozierten otoakustischen Emissionen (TEOAE) untersucht. Bei Auffälligkeiten erfolgt eine zweite Untersuchung, häufig mit automatisierter Hirnstammaudiometrie (BERA). Für Säuglinge mit Risikofaktoren wird die BERA als primäres Instrument zur Erkennung einer potenziellen auditorischen Neuropathie empfohlen, die mit TEOAE nicht erkannt würde. Diese Protokolle ermöglichen eine frühzeitige Diagnose und Behandlung, wodurch das Potenzial der Kinder, eine altersgerechte gesprochene Sprache zu entwickeln, maximiert und gleichzeitig langfristiger Bedarf an speziellen Bildungsangeboten gesenkt wird.

Früherkennung wird immer wichtiger, da die therapeutischen Möglichkeiten für angeborenen Hörverlust über herkömmliche Geräuschverstärkung zunehmend hinausgehen. Für Kinder mit schwerem bis hochgradigem SNHV ist die Cochleaimplantation eine etablierte Intervention. Es besteht starke Evidenz, dass eine frühzeitige Implantation (im Idealfall innerhalb des ersten Lebensjahres) sicher ist und zu überlegenen Sprach‑, Sprech- und kognitiven Ergebnissen führt, indem sie die Phase maximaler neuronaler Plastizität nutzt [15, 17]. Aktuelle Daten unterstreichen zudem die Vorteile einer simultanen bilateralen Versorgung, welche im Vergleich zum sequenziellen Vorgehen die Narkose- und Operationszeit reduziert, ohne die perioperative Morbidität zu erhöhen [8].

Zunehmende klinische Relevanz gewinnen auch die einseitige Taubheit („single-sided deafness“, SSD) und der asymmetrische Hörverlust (AHL) im Kindesalter. Unbehandelt drohen Defizite in der binauralen Verarbeitung und der schulischen Entwicklung. Langzeitstudien belegen hier, dass eine frühzeitige Cochleaimplantat(CI)-Versorgung – im Idealfall vor dem dritten Lebensjahr – zu stabilen Hörergebnissen, verbessertem Sprachverstehen im Störschall und einer hohen Akzeptanz führt [1, 24].

Gentherapie entwickelt sich zu einer Behandlungsmöglichkeit für genetische Schwerhörigkeit

Darüber hinaus zeichnet sich ein Paradigmenwechsel von der neuroprothetischen Rehabilitation hin zur biologischen Wiederherstellung ab. Mit über 150 bekannten Genen, die mit nichtsyndromalem Hörverlust in Verbindung stehen, entwickelt sich die Gentherapie zu einer Behandlungsmöglichkeit für bestimmte Formen der genetischen Schwerhörigkeit [16]. Bahnbrechende klinische Phase-I/II-Studien haben bemerkenswerte Erfolge bei der Wiederherstellung des Hörens bei Kindern mit angeborener Taubheit aufgrund biallelischer Funktionsverlustmutationen im *OTOF*-Gen gezeigt [16]. Otoferlin ist essenziell für die kalziumabhängige Exozytose synaptischer Vesikel in den inneren Haarzellen. Ein Defekt führt zu einer auditiven Synaptopathie bei morphologisch intakten Haarzellen, was diese seltene Form der Schwerhörigkeit (etwa 1–8 % der hereditären nichtsyndromalen Fälle) zu einem idealen Ziel für den Genersatz macht. Diese neuen Behandlungen bestehen aus nur einer einzigen Applikation in die Cochlea, um eine funktionsfähige Kopie des Gens zu übertragen und eine stabile Expression zu erreichen. Während diese Erfolge einen historischen Machbarkeitsnachweis darstellen, ist die Übertragung auf andere genetische Ursachen herausfordernd. Ansätze für die weitaus häufigeren Connexin-Gendefekte (*GJB2*) befinden sich derzeit überwiegend noch in präklinischen Tiermodellen. Da Connexine breit in Stützzellen exprimiert werden, ist die Gentherapie hier deutlich komplexer. Es ist derzeit noch unklar, in welchem Ausmaß sich durch diese zukünftigen Therapieansätze tatsächlich ein differenziertes Sprachverstehen ausbilden kann. Trotzdem unterstreicht diese Entwicklung eine zukünftige Konvergenz der Diagnostik, bei der ein fehlgeschlagenes UNHS wahrscheinlich auch zeitnahe Gentests erforderlich machen wird, um die Wahl zwischen technologischen Eingriffen wie einem CI und biologischer Korrektur durch Gentherapie zu ermöglichen [5].

Während sich diese fortschrittlichen Therapien derzeit auf die Behandlung von angeborenem Hörverlust konzentrieren, sind etablierte Strategien im Bereich der öffentlichen Gesundheit unerlässlich, um die viel häufigeren erworbenen Formen zu verhindern. Routinemäßige Impfungen im Kindesalter stellen eine der wirksamsten Maßnahmen dar [11]. Mehrere Krankheiten, die häufige Ursachen für Taubheit waren, werden heute weitgehend durch Standardimpfstoffe eingedämmt, wie in Tab. 2 dargestellt. Der MMR-Impfstoff schützt beispielsweise sowohl vor Masern, die über Enzephalitis zu SNHV führen können, als auch vor Mumps, einer klassischen Ursache für einseitigen SNHV. Darüber hinaus waren vor der Entwicklung der entsprechenden Impfstoffe *Haemophilus influenzae* Typ B (HiB), *Streptococcus pneumoniae* (Pneumokokken) und *Neisseria meningitidis *(Meningokokken) Erreger bakterieller Meningitis. Diese birgt ein hohes Risiko für schweren SNHV und nachfolgende Labyrinthitis ossificans, die eine CI-Operation unmöglich machen kann [11, 15]. Zwar können alle 3 genannten Erreger (Pneumokokken, HiB und Meningokokken) zu einer Verknöcherung der Cochlea führen, klinisch ist dies jedoch besonders häufig und rasch nach einer Pneumokokkenmeningitis zu beobachten. Um eine spätere CI-Versorgung nicht durch eine Obliteration zu gefährden, sind bei postmeningitischem Hörverlust eine engmaschige BERA-Kontrolle sowie die frühzeitige Durchführung einer Magnetresonanztomographie (MRT, insbesondere T2-gewichtete CISS-Sequenzen) von großer klinischer Relevanz.

HiB und Pneumokokken sind auch die häufigsten Ursachen der Otitis media

HiB und Pneumokokken sind auch die häufigsten Ursachen der Otitis media, die zu schweren Komplikationen wie chronischer suppurativer Otitis media und direkten Innenohrschäden führen kann [11]. Daher sind die jeweiligen Impfungen nicht nur für die Vorbeugung systemischer Erkrankungen von entscheidender Bedeutung, sondern auch für die Erhaltung des Hörvermögens und die Durchführbarkeit einer zukünftigen auditorischen Rehabilitation.

**Tab. 2:** Impfungen zur Vorbeugung von Hörverlust.

Erreger/Krankheit	Impfstoff	Ursache des SNHV	Zielgruppe und Zeitpunkt
Röteln	MMR	Angeborenes Rötelnsyndrom	*Kinder*: 2 Dosen empfohlen. 1. Dosis im Alter von 11 Monaten (DE)/ab Vollendung des 9. Monats (AT); 2. Dosis im Alter von 15 Monaten (DE)/mindestens 3 Monate nach der 1. Dosis (AT)
			*Erwachsene*: Bei Personen, die nach 1970 geboren wurden oder deren Status unklar ist, sollten 2 Dosen dokumentiert werden
Masern	MMR	Masernenzephalitis	*Wie oben ausgeführt*
Mumps	MMR	Mumpsassoziierte virale Labyrinthitis	*Wie oben ausgeführt*
*Streptococcus pneumoniae* (Pneumokokken)	Pneumokokken-Konjugatimpfstoff (PCV)	Pneumokokkenmeningitis	*Säuglinge* (Standard): 2 + 1-Schema. Dosen im Alter von 2, 4 und 11 Monaten (DE, PCV13/15 empfohlen); Dosen im Alter von 6 Wochen, 8 Wochen später und 6 Monate nach der 2. Dosis (AT, PCV15-kostenloses Programm)
			*Frühgeborene: *3 + 1-Schema, d. h. zusätzliche Dosis im Alter von 3 Monaten (DE & AT)
*Haemophilus influenzae* Typ B	HiB-Impfstoff (i. d. R. als Teil eines Sechsfachimpfstoffs)	HiB-Meningitis	*Säuglinge*: 2 + 1-Schema. Dosen im Alter von 2, 4 und 11 Monaten (DE); Dosen nach Abschluss der 6. Woche, 2 Monate später und 6 Monate nach der 2. Dosis (AT). Nur empfohlen bis zum Abschluss des 5. Lebensjahres, sofern keine spezifische Indikation vorliegt
*Neisseria meningitidis* (Meningokokken)	Meningokokken-Konjugatimpfstoffe (MenB, MenC/ACWY)	Meningokokkenmeningitis	*MenB*: Standard für Säuglinge im Alter von 2, 4 und 12 Monaten (DE); empfohlen für Säuglinge und Jugendliche (gleicher 2 + 1-Impfplan ab ≥ 2 Monaten), jedoch kein kostenloses Programm (AT)
			*MenC: *Standard für Kinder im Alter von 12 Monaten (DE)
			*MenACWY: *Indikationsbasiert (DE); empfohlen für Kleinkinder (~13 Monate) und Kinder/Jugendliche (10–13 Jahre), 2. Dosis im Rahmen eines kostenlosen Programms (AT)

*AT* Österreich, *DE* Deutschland, *MenACWY *Meningokokken der Serogruppen A, C, W, Y

## Prävention im Erwachsenenalter

Obwohl Geburt und frühe Kindheit eine entscheidende Rolle spielen, tritt Hörverlust überwiegend im Erwachsenenalter auf und ist größtenteils auf kumulative Umwelteinflüsse und Lebensstilfaktoren zurückzuführen. Im Gegensatz zum UNHS ist die routinemäßige Höruntersuchung bei asymptomatischen Erwachsenen jedoch nach wie vor umstritten. In Österreich ist ein Hörtest fester Bestandteil der jährlichen Vorsorgeuntersuchung, was die Früherkennung von Hörverlust, insbesondere der Presbyakusis, erleichtert. In Deutschland ist dies jedoch nicht der Fall, was zu einer geringeren Diagnoserate beitragen könnte [4]. Dennoch sind verfügbare Screeningangebote ein wertvolles Instrument für Erwachsene mit identifizierbaren Risikofaktoren wie Diabetes mellitus, vorangegangener Lärmbelastung oder der Einnahme ototoxischer Medikamente.

Von diesen Risikofaktoren ist übermäßige Lärmbelastung – sowohl am Arbeitsplatz als auch in der Freizeit – eine der bedeutendsten und häufigsten, aber gleichzeitig auch vermeidbarsten Ursachen von SNHV im Erwachsenenalter. Präventionsstrategien umfassen eine Hierarchie von Kontrollmaßnahmen: technische Schritte zur Reduzierung des Lärms an der Quelle, administrative Mittel wie die Begrenzung der Expositionszeit und die konsequente Verwendung von persönlichem Gehörschutz [26]. Die Sicherheit am Arbeitsplatz wird durch nationale Vorschriften geregelt, während Freizeitlärm ein zunehmendes Gesundheitsproblem in der Bevölkerung, v. a. bei Jugendlichen, darstellt. Ärzte spielen eine entscheidende Rolle bei der Aufklärung über sichere Hörgewohnheiten [28].

Ein weiteres häufiges Problem ist medikamenteninduzierter Hörverlust. Ärzte müssen das therapeutische Bedürfnis gegen das ototoxische Risiko abwägen und von reaktiver Behandlung zu einer proaktiven Risikominderung übergehen. Am häufigsten sind hierbei platinbasierte Chemotherapeutika und Aminoglykosid-Antibiotika [25]. Cisplatin beispielsweise verursacht oft irreversiblen, beidseitigen, hochfrequenten SNHV, insbesondere bei pädiatrischen Patienten. Die Prävention erfordert einen multidisziplinären Ansatz, einschließlich serieller Audiometrien. Ein bedeutender Fortschritt ist die Zulassung von Natriumthiosulfat, das zur Risikominderung der Cisplatin-induzierten Ototoxizität bei Kindern mit lokalisierten, nichtmetastasierten soliden Tumoren indiziert ist [2]. Der Zusammenhang zwischen dem Gehör und weiteren Medikamenten wie Statinen ist komplex. Einige Hinweise deuten auf eine mögliche protektive Wirkung von Statinen gegen Cisplatin-induzierten SNHV hin, möglicherweise aufgrund antioxidativer und entzündungshemmender Eigenschaften [6]. Andererseits wurde auch ein Zusammenhang zwischen Statineinnahme (v. a. Simvastatin) und einer höheren Prävalenz von Hörverlust festgestellt, was neue Fragen aufwirft [27].

Cisplatin-Ototoxizität kann durch Natriumthiosulfat gemildert werden

Darüber hinaus gibt es zunehmend Hinweise für den Zusammenhang veränderbarer Lebensstilfaktoren mit der Hörfunktion. Tabakrauchen ist ein bedeutender, dosisabhängiger Risikofaktor für Hörverlust, wobei als Mechanismen u. a. direkte Ototoxizität durch Nikotin sowie vasokonstriktorische Innenohr-Ischämie vermutet werden [3]. Die Beratung zur Raucherentwöhnung ist hier eine wirksame Präventionsstrategie. Darüber hinaus kann starker Alkoholkonsum den auditiven Kortex und die Haarzellen schädigen und erhöht somit ebenfalls das Risiko [3]. Im Gegensatz dazu kann ein gesunder Lebensstil mit einer ausgewogenen Ernährung, die reich an Antioxidanzien, Mineralien und Omega-3-Fettsäuren ist, sowie regelmäßiger körperlicher Aktivität dazu beitragen, das Gehör zu erhalten, indem eine gute Mikrozirkulation der Cochlea gefördert und oxidativer Stress reduziert wird [29]. Die Behandlung systemischer vaskulärer Risikofaktoren sowie regelmäßiger Kaffeekonsum sind aus ähnlichen Gründen wahrscheinlich ebenfalls von Vorteil. Darüber hinaus ist die Aufklärung der Patienten über grundlegende Ohrhygiene, wie z. B. die Vermeidung von Wattestäbchen zur Reinigung des äußeren Gehörgangs, eine einfache, aber wirksame Maßnahme, um Cerumen obturans, Otitis externa und sogar Trommelfellperforationen zu verhindern.

## Prävention der Folgen von Hörverlust

Bei Personen mit nachgewiesenem, irreversiblen Hörverlust verlagert sich der Schwerpunkt auf Tertiärprävention, also die Abschwächung der Folgeerscheinungen auf Kognition, soziale Integration und allgemeine Gesundheit. Eine Vielzahl epidemiologischer Belege zeigte, dass Hörverlust einer der größten unabhängigen und potenziell veränderbaren Risikofaktoren für einen beschleunigten kognitiven Verfall ist [23]. Tatsächlich identifizierten die Lancet-Kommissionen zur Demenzprävention 2020 und 2024 Hörverlust im mittleren Lebensalter als den größten veränderbaren Risikofaktor, welcher schätzungsweise 8 % aller Demenzfälle ausmacht [19]. Es gibt mehrere Hypothesen zu den Mechanismen, die diesem Zusammenhang zugrunde liegen. Erstens belastet ein verschlechtertes Hörsignal, bei dem das Gehirn mehr Kapazitäten für dessen Entschlüsselung aufwenden muss („angestrengtes Hören“), andere kognitive Systeme und lässt weniger Ressourcen für das Gedächtnis und die exekutiven Funktionen übrig. Ein zweiter Faktor sind Veränderungen in der Gehirnstruktur, da sensorische Deprivation langfristig zu einer beschleunigten Hirnatrophie in den auditorischen Verarbeitungszentren und anderen damit verbundenen Regionen führen kann [21, 23]. Darüber hinaus führen kommunikative Schwierigkeiten oft zu sozialem Rückzug, verminderter Teilnahme an kognitiv stimulierenden Aktivitäten und Depression, welche allesamt selbst unabhängige Risikofaktoren für Demenz sind [23]. Untersuchungen konnten zudem zeigen, dass Hörstörungen mit einer erhöhten Prävalenz von Angststörungen und psychosozialen Komorbiditäten assoziiert sind. Eine auditive Rehabilitation – sei es bei beidseitigem Hörverlust oder bei SSD/AHL – führt dabei nicht nur zu einer Verbesserung des Hörens, sondern reduziert signifikant Tinnitusbelastung sowie psychische Begleitsymptome und steigert die gesundheitsbezogene Lebensqualität [10, 22].

Dieser starke Zusammenhang erhebt auditive Rehabilitation von einer Maßnahme zur Verbesserung der individuellen Lebensqualität zu einer wesentlichen Strategie im Bereich der öffentlichen Gesundheit. So ergab beispielsweise die richtungsweisende ACHIEVE-Studie, dass die Verwendung von Hörgeräten über einen Zeitraum von 3 Jahren den kognitiven Verfall in der Teilnehmergruppe mit einem höheren Ausgangsrisiko für Demenz um 48 % verlangsamte [18]. Dies zeigt, dass die frühzeitige Anpassung und konsequente Verwendung von Hörgerät oder CI nicht nur die Lebensqualität verbessert, sondern auch die langfristige Gesundheit des Gehirns unterstützt.

## Fazit für die Praxis

- Wichtig ist die Beratung werdender Mütter bzgl. Impfungen und Hygienemaßnahmen zur Vorbeugung angeborener Infektionen, z. B. mit dem Zytomegalievirus (CMV).
- Ein auffälliges Ergebnis beim universellen Neugeborenen-Hörscreening (UNHS) sollte eine audiologische Nachuntersuchung und ggf. gezielte CMV-Tests nach sich ziehen, um eine rechtzeitige Intervention in kritischen Entwicklungsphasen zu ermöglichen.
- Die Kontrolle von Routineimpfungen dient dazu, Hörverlust durch Virusinfektionen und bakterielle Meningitis zu verhindern.
- Beratungen zu Lärmschutz, sicheren Hörgewohnheiten und allgemeinem Lebensstil sind zu empfehlen.
- Ein Basismonitoring und die serielle audiometrische Überwachung bei Cisplatin-Therapie und ggf. Natriumthiosulfat (zzt. nur bei pädiatrischen Patienten) sollten erfolgen.
- Aktive Vorsorgeuntersuchungen bei Patienten mit Hörverlust und rechtzeitige Förderung auditiver Rehabilitation sind sinnvoll, um das Risiko von sozialer Isolation, Depression und kognitivem Abbau zu verringern.
